# Deep robust residual network for super-resolution of 2D fetal brain MRI

**DOI:** 10.1038/s41598-021-03979-1

**Published:** 2022-01-10

**Authors:** Liyao Song, Quan Wang, Ting Liu, Haiwei Li, Jiancun Fan, Jian Yang, Bingliang Hu

**Affiliations:** 1 The school of information and communications engineering, Xi’an Jiaotong University, Xi’an, 710049 China; 2 Xi’an Institute of Optics and Precision Mechanics of Chinese Academy of Sciences, Xi’an, 710049 China; 3 The First Affiliated Hospital of Xi’an Jiaotong University, Xi’an, 710061 China

**Keywords:** Computational biology and bioinformatics, Computational models, Data processing

## Abstract

Spatial resolution is a key factor of quantitatively evaluating the quality of magnetic resonance imagery (MRI). Super-resolution (SR) approaches can improve its spatial resolution by reconstructing high-resolution (HR) images from low-resolution (LR) ones to meet clinical and scientific requirements. To increase the quality of brain MRI, we study a robust residual-learning SR network (RRLSRN) to generate a sharp HR brain image from an LR input. Due to the Charbonnier loss can handle outliers well, and Gradient Difference Loss (GDL) can sharpen an image, we combined the Charbonnier loss and GDL to improve the robustness of the model and enhance the texture information of SR results. Two MRI datasets of adult brain, Kirby 21 and NAMIC, were used to train and verify the effectiveness of our model. To further verify the generalizability and robustness of the proposed model, we collected eight clinical fetal brain MRI 2D data for evaluation. The experimental results have shown that the proposed deep residual-learning network achieved superior performance and high efficiency over other compared methods.

## Introduction

Spatial resolution is a key factor of evaluating the quality of magnetic resonance imaging (MRI). Images having high spatial resolution produce rich structural details, enabling accurate image analysis and detailed anatomical information for accurate quantitative analysis^1^. The recent development of fast MRI slice acquisition techniques has enabled MRI to be used for fetal imaging. MRI can be used to assess brain disease and diagnose fetal congenital brain malformations. High-quality and HR slices can be obtained through fast slice acquisition techniques such as half-Fourier acquisition single shot fast spin echo (SSFSE)^2^. The slices are acquired as snapshots in fractions of a second, thus freezing the motion of the subject. Therefore, MRI is one of examination methods for prenatal screening and has a broad application prospect. Although high-quality slices are frequently acquired by these techniques, due to the interference of amniotic fluid, placenta, maternal pelvis, and fetal skull, limitations of the equipment’s component performance, fetal motion and other factors, fetal brain slices can’t reach the quality of neonatal imaging. Especially, MRI needs to be done quickly to avoid motion artifacts, one way to speed up is to acquire the lower resolution image. Overall, the above limitations of the component performance of equipment, uncooperative patients, and other factors, improvements to 2D MRI quality are necessary^3^.

**Figure 1:**
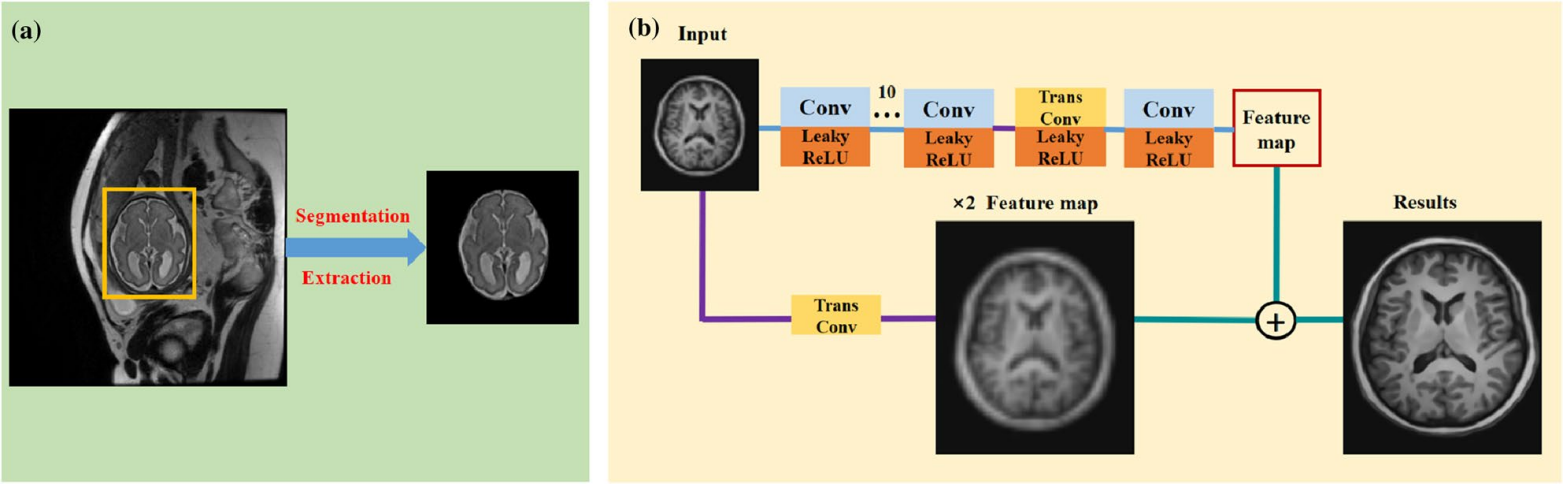
(**a**) When we use fetal data, we label and segment fetal brains under professional guidance. (**b**) The proposed RRLSRN architecture for brain MRI SR.

With conventional medical image processing, bicubic or spline interpolation is usually adopted as standard image-processing techniques to be more convenient to match the resolution of internal atlases for a volume input with thicker slices. This interpolation method negatively affects image accuracy^4^. Therefore, coherently recovering the missing information during the acquisition of medical images and better reconstructing the high-resolution (HR) image is a fundamental problem in the field.

Convolutional neural networks (CNN) have been widely used for natural images, and CNN-based super-resolution (SR) algorithms have been extended to MRI^5–18^. Many SR algorithms are based on SR combined with CNNs (SRCNN). Zeng et al.^12^ proposed a model that simultaneously performed single- and multi-contrast SR reconstruction. To capture the cubic spatial feature of the MRI, Du et al.^11^ exploited 3D dilated convolution as encoder to extract high-frequency features, resulting in good performance. Based on this model, Pham et al.^6^ developed a SRCNN algorithm which employed 3D covolutions for brain MRI SR, and the network performed excellently.

The input to above SRCNNs must be a bicubic low-resolution (LR) image. To reduce the computational cost, Fast SRCNN^19^ adopted a deconvolutional layer to reconstruct HR images from LR features. Shi et al.^20^ proposed an efficient sub-pixel CNN. When the redundant nearest-neighbor interpolation was replaced with the interpolation, the deconvolutional layer was simplified into a sub-pixel convolution. This interpolation was more efficient than the nearest-neighbor interpolation.

**Figure 2:**
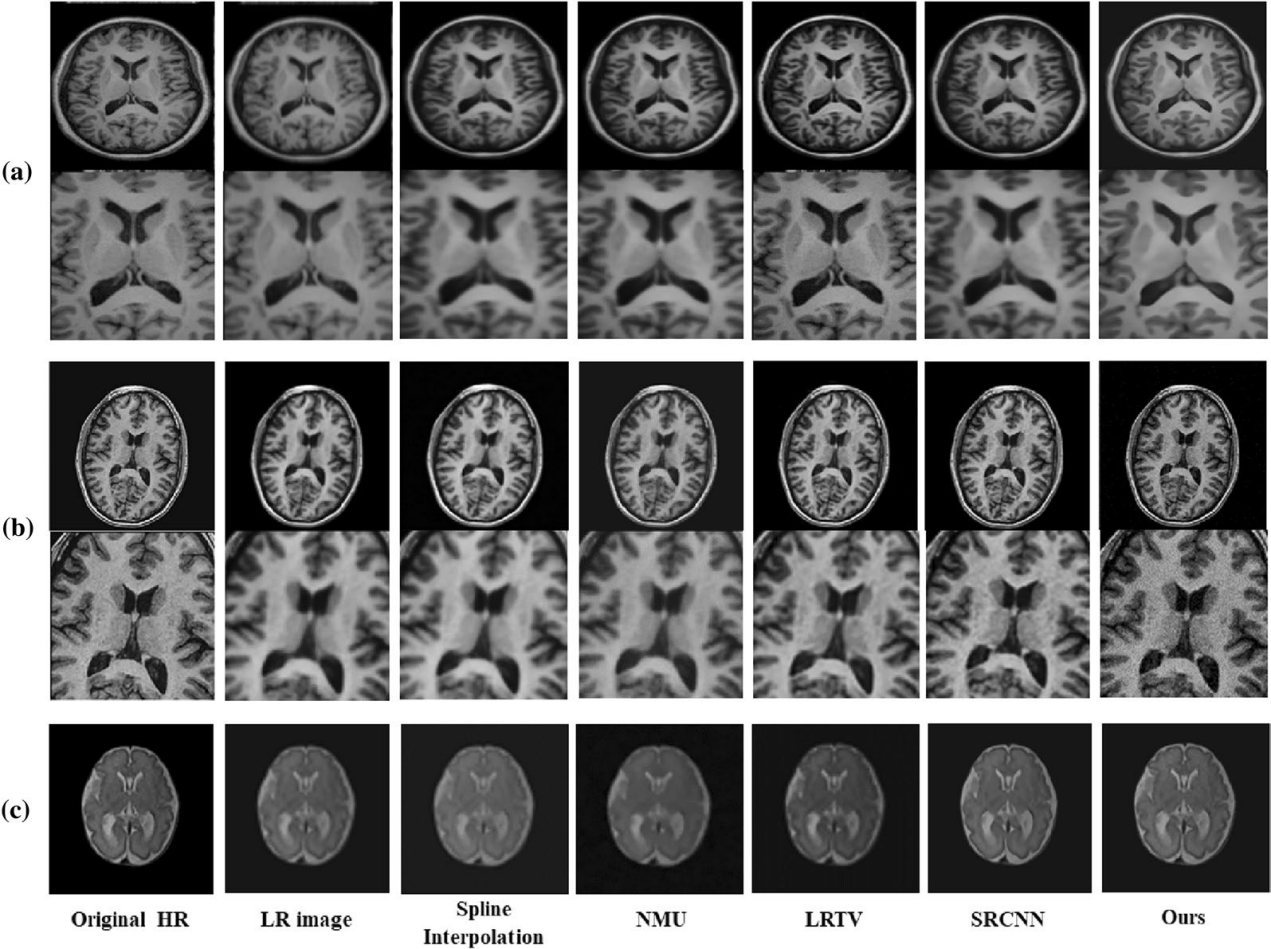
Illustration of SR results with upsampling (scale factor is 2): (**a**) Kirby 21; (**b**) NAMIC; (**c**) clinical fetal brain MR images.

**Figure 3:**
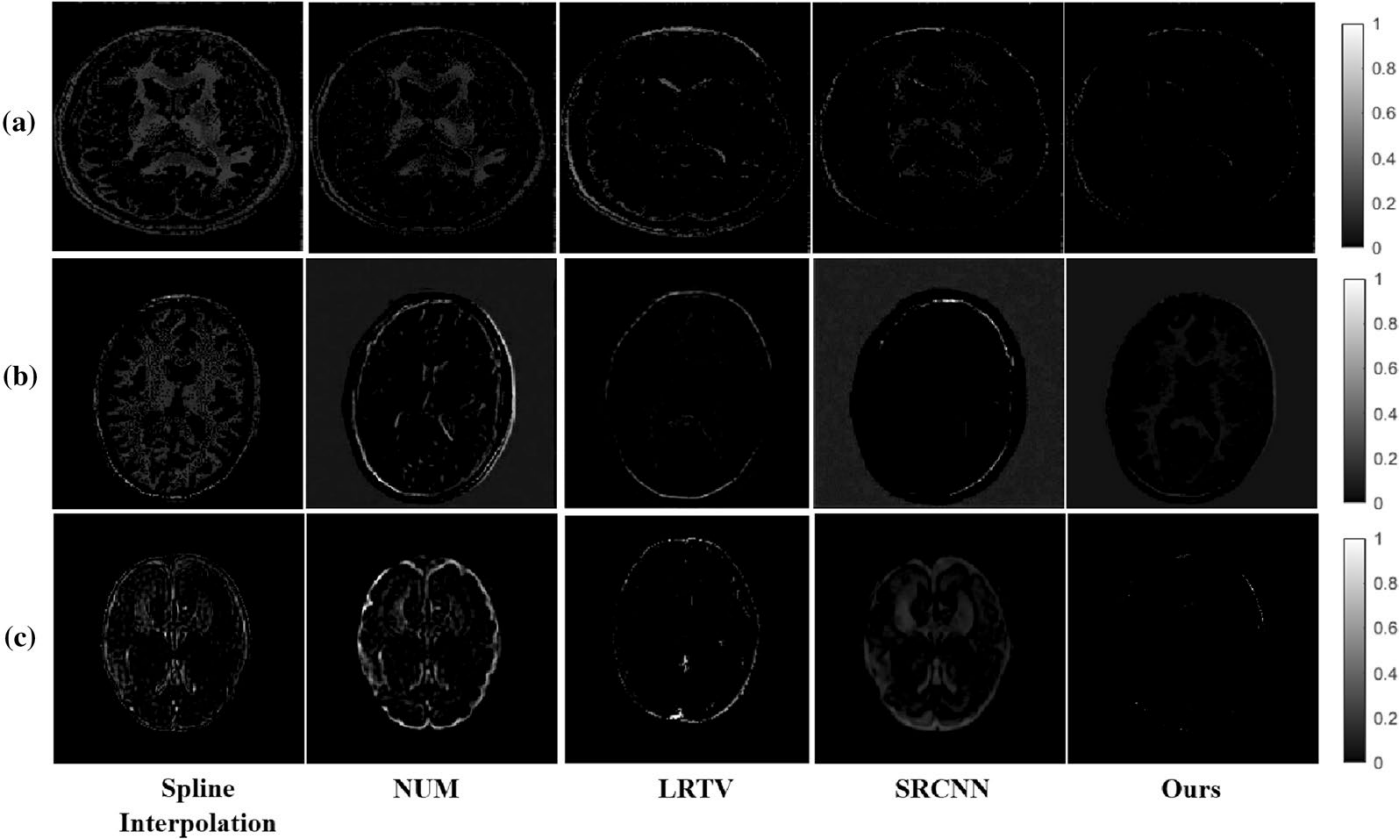
The error maps of SR results: (**a**) Kirby 21; (**b**) NAMIC; (**c**) clinical fetal brain MR images.

**Table 1:** The mean, standard deviation (SD) and confidence interval (CI) of PSNR/SSIM for scale factor $\times 2$ between our method and compared methods on Kirby 21 dataset.

Kirby 21	Metric	Cubic spline	NMU	LRTV	SRCNN	Ours
Mean	PSNR	34.16	34.40	35.26	36.56	37.16
SD	PSNR	1.90	2.00	1.90	1.02	1.05
CI (95%)	PSNR	[31.80,36.51]	[31.87,36.87]	[32.90,37.62]	[35.29,37.83]	[35.90,38.43]
Mean	SSIM	0.9402	0.9464	0.9589	0.9496	0.9902
SD	SSIM	0.1109	0.1056	0.0083	0.0088	0.0013
CI (95%)	SSIM	[0.9264,0.9540]	[0.9333,0.9595]	[0.9485,0.9692]	[0.9388,0.9605]	[0.9886,0.9919]

**Table 2:** The mean, standard deviation (SD) and confidence interval (CI) of PSNR/SSIM for scale factor $\times 2$ between our method and compared methods on NAMIC dataset.

NAMIC	Metric	Cubic spline	NMU	LRTV	SRCNN	Ours
Mean	PSNR	33.78	28.68	34.34	33.26	35.56
SD	PSNR	1.83	0.64	1.79	0.78	0.34
CI (95%)	PSNR	[31.51,36.05]	[27.88,29.48]	[32.12,36.56]	[32.29,34.24]	[35.14,35.99]
Mean	SSIM	0.9388	0.5590	0.9549	0.9447	0.9821
SD	SSIM	0.0069	0.0134	0.0044	0.0049	0.0040
CI (95%)	SSIM	[0.9303,0.9473]	[0.5430,0.5762]	[0.9488,0.9595]	[0.9388,0.9598]	[0.9765,0.9896]

**Table 3:** The mean, standard deviation (SD) and confidence interval (CI) of PSNR/SSIM for scale factor $\times 2$ between our method and compared methods on clinical fetal brain MRI dataset.

Fetal brain MRI	Metric	Cubic spline	NMU	LRTV	SRCNN	Ours
Mean	PSNR	33.61	32.63	34.78	35.91	39.40
SD	PSNR	2.08	2.44	2.10	2.84	0.33
CI (95%)	PSNR	[31.03,36.19]	[29.61,35.67]	[32.17,37.39]	[32.38,39.44]	[38.99,39.81]
Mean	SSIM	0.9983	0.9546	0.9913	0.9564	0.9897
SD	SSIM	0.0009	0.0336	0.0019	0.0045	0.0001
CI (95%)	SSIM	[0.9972,0.9994]	[0.9505,0.9588]	[0.9897,0.9942]	[0.9507,0.9620]	[0.9896,0.9898]

Although these models demonstrated promising results, they all required upscaled input images at the desired spatial resolutions via bicubic interpolation prior by applying the network, and these models did not use low-level feature information. To cope with these limitations, some SR algorithms have adopted residual learning^5,7–9,13,21,22^, showing effective improvements.

In this work, there are three aspects of our contributions: (1) To address the computational-cost problem and avoid generating fake features, we adopted a deep residual network to train residuals in a coarse-to-fine fashion. (2) In order to sharpen the SR image, we combined Gradient Difference Loss (GDL)^23^ and the robust Charbonnier loss function, this way can deal with outliers and improve reconstruction accuracy. (3) We collected eight clinical fetal-brain MRIs for further evaluating the generalizability and robustness of the proposed model.

## Experimental results

Figure 2 has shown the HR example slices for the different algorithms: cubic spline interpolation and non-local means up-sampling (NMU)^24^ , low-rank total variation (LRTV)^25^, and SRCNN^26^ for visual inspection with the ground-truth MR image and LR image on Kirby 21, NAMIC^1^, and clinical fetal MR images, respectively. All the figures in our paper were drawn by Microsoft Office PowerPoint 2016 (https://www.office.com/). It can be seen that our approach recovered fine details and preserved the edges.

The SR deep-learning technique was not very limited by MRI parameters and could therefore be further migrated to the fetal brain. Thus, we applied our model to fetal MRIs, which were provided by the First Affiliated Hospital of Xi’an Jiaotong University. We labeled the fetal brain on the MRI and extract the fetal brain. The MRIs of each fetus were cut into 10–20 slices. We tested all slices of each fetus. Figure 2c shows the SR example slices of different algorithms on a subject. The reconstructed MR images by our network provided more details than did the other algorithms. The error maps Fig. 3 can make it easier to identify differences between the methods.

For a quantitative comparison, the average peak signal-to-noise ratio and structural similarity^27^ were used to evaluate the performance of each algorithm. Tables 1, 2 and 3 provided a summary of the quantitative evaluation within a scale factor of two, include Mean, Standard Deviation (SD) and confidence interval (CI) which confidence level is $95\%$of PSNR and SSIM. The reported results tend to show that CNN-based approaches (e.g., SRCNN and our RRLSRN model) achieved better performance than did cubic spline, NMU, and LRTV. Our experiments also showed that residual learning approaches were more effective than SRCNN.

In our model, we combined the Charbionner loss and GDL to train our model. To verify the effect of GDL on SR results, we compared the PSNR of model without GDL on 8 clinical fetal brain MR images, the results are shown as Table 4. All PSNR of 8 fetal MR images with the GDL are higher than without GDL. The results demonstrate that GDL is helpful to improve the quality of images.

**Table 4:** The PSNR results compared with/without GDL.

ID	01	02	03	04	05	06	07	08
Without GDL	39.03	38.66	40.24	39.83	39.54	39.39	38.79	38.71
With GDL	39.18	38.68	40.36	39.95	39.64	39.54	38.96	38.90

**Figure 4:**
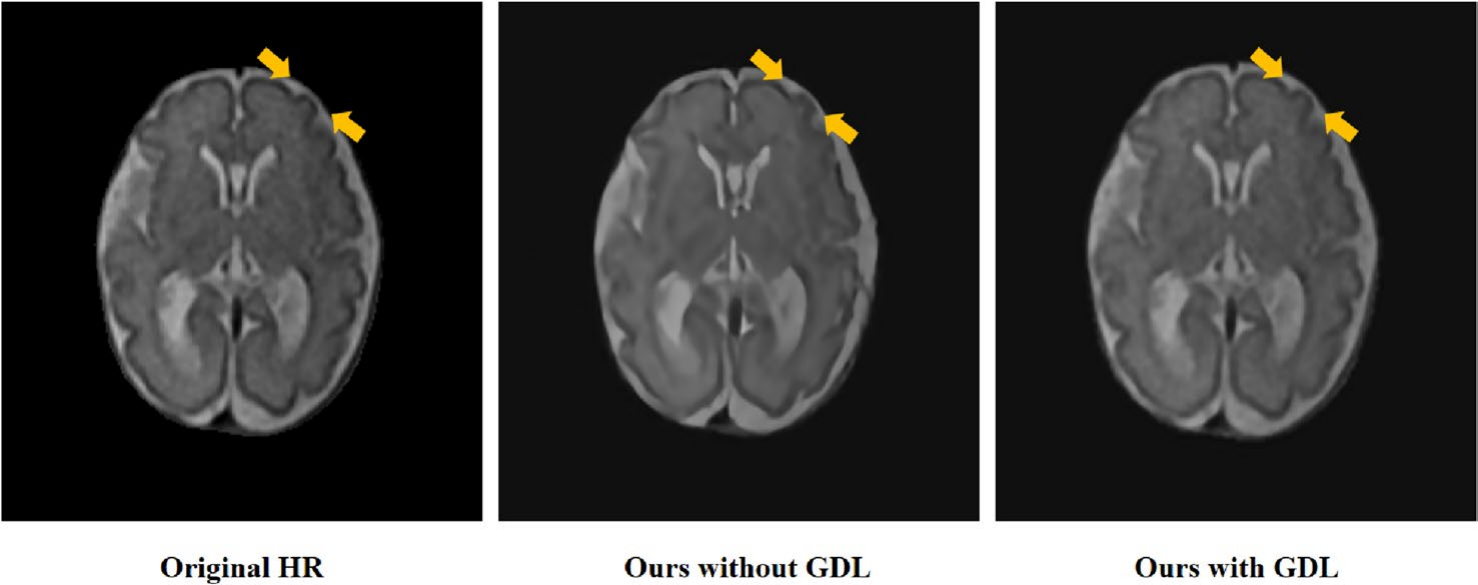
Visual difference between our model with GDL and without GDL on the clinical fetal brain MRI dataset.

Our experiment has shown that the proposed model with GDL can enhance the brain’s edge of MRI. And we show the visual difference between our model with GDL and without GDL on the clinical fetal brain MRI dataset as Fig. 4. As shown by the yellow arrow , the reconstruction result of our model with GDL has sharper edges and is similar with HR image than the model without GDL.

We trained the model without the transpose convolution at the bottom of our model to demonstrate the effect of transpose convolution. We compared the PSNR on 8 clinical fetal brain MR images, the results have been shown as Table 5. The experimental results show that transpose convolution at the bottom is helpful to improve the accuracy of the results. Residual learning is beneficial to the model.

**Table 5:** The PSNR results compared with/without transpose convolution of bottom.

ID	01	02	03	04	05	06	07	08
Without transpose convolution of bottom	36.78	35.89	38.11	37.89	36.58	37.77	36.24	35.92
With transpose convolution of bottom	39.18	38.68	40.36	39.95	39.64	39.54	38.96	38.90

**Table 6:** Comparison of computational speed (second) with different methods.

Dataset	Cubic Spline	NMU	LRTV	SRCNN (faster version)	Ours
Kirby 21	0.0104	6.5719	11.7059	1.7473	0.8244
NAMIC	0.0128	10.3675	6.9668	1.5395	0.8020
Fetal MRI	0.0125	8.5935	9.7356	1.7254	0.8173

To verify the efficiency of our algorithm, we separately calculated the test time of our Kirby 21, NAMIC, and the fetal MR image methods. We then compared the spending time of other methods. The results are shown in Table 6. The average speed of our model was faster than those of the NMU, LRTV, SRCNN (faster version)^19^ on three datasets.

## Discussion

In this work, we proposed a network-based algorithm to learn the residual information between upsampled MR images and HR MR images. Our approach adpoted the robust Charbonnier loss function and GDL which are helpful to train our model. In order to demonstrate the potential of SR methods for enhancing the quality of LR images, we have presented an experiment with image quality transfer from HR experimental dataset to LR images. The results based on two brain MR image datasets have shown that our algorithm outperforms cubic spline, NMU, LRTV and SRCNN in this study. RRLSRN network effectively learned the residual information between upsampled LR MRI and HR MRI, the model can not only improve the accuracy of network SR results, but also greatly reduce the computational cost. Then we applied the model on the clinical fetal MR images. The fetal SR results of the proposed RRLSRN are better than above listed methods. The texture of SR results become detailed.

In terms of the processing speed, we observed that our method trained $\times 2$ faster than NMU, LRTV and SRCNN on both Kirby 21 and NAMIC datasets. Overall, our algorithm performed well in terms of speed.

Our SR method has shown clear improvement over other listed methods, which is the standard technique to enhance image quality from visualization, quantitative evaluation and computational efficiency. Our model is currently SR on the scale of $\times 2$ of 2D MR slices, it can also be extended to $\times 4$ or $\times 8$ times for SR reconstruction by cascading. In future work, we will improve our residual learning based SR framework to obtain better accuracy, meanwhile reduce computational complexity. In addition, we will further apply the SR technology to improve the accuracy and validity of the clinical diagnosis by combining the equipment.

## Methods

### MR image super-resolution framework

We proposed RRLSRN to generate an HR brain image from its LR input. Our network is made up of the feature extraction and image reconstruction parts. The image reconstruction part estimates a raw HR output and extracts useful representations from LR MRI. We up-sampled LR MRI and learned the residual information between the HR MRI and the up-sampled MRI. Our LR MRI is derived from the HR MRI via bicubic interpolation.

1 $$\begin{aligned} r=&y-(u(\kappa B y)) \nonumber \\&=y-u x \nonumber \\&=y-z \end{aligned}$$

where *x* and *y* represent the LR and HR images, respectively. $\kappa$ is the down-sampling operator. *r* is the residual information between the HR MRI and the bicubic-interpolated MRI. *u* represents the up-sampling operator. The model can learn the residual feature and up-sampling feature with normal and transposed convolutional layers. The network architecture used in this study is illustrated in Fig. 1b. When using fetal data, we segmented and extracted fetal brains as shown Fig. 1a.

The main architecture of the network for feature extraction consisted of 13 convolutional layers and two transposed convolutional layers to up-sample the extracted features using a scale of two. Because the fetal MRI slice sequence did not enable 3D representation, we designed our model with 2D convolution. The convolution kernel size was $3 \times 3 \times 64$. The transpose convolutions were $4 \times 4 \times 1$. Our model performed feature extraction at a coarse resolution and generated feature maps with finer details by using the transposed convolutional layer. Compared to the listed networks, our network can reduce computational complexity significantly.

### Loss function

This approach can learn the information lost in the image by interpolation, and it can also reduce computational complexity. We optimized the network with a Charbonnier loss^4^, as stated in the following formulation:

2 $$\begin{aligned} L_{Charbonnier}(y, \widehat{y})&=\sqrt{x^{2}+\varepsilon ^{2}}(\hat{y}-y) \nonumber \\&=\sqrt{x^{2}+\varepsilon ^{2}}(\hat{y}-(u x+r)) \nonumber \\&=\sqrt{x^{2}+\varepsilon ^{2}}((\hat{y}-u x)-r) \end{aligned}$$

Let *x* be the input. We denote the ground-truth HR MRI slice by *y*, generating the corresponding HR MRI slice by $\hat{y}$, and the residual information of MRI by *r*. The overall Charbonnier loss function is:

3 $$\begin{aligned} L_{Charbonnier}(y_s, \hat{y}_s)&=\frac{1}{N} \sum _{s=1}^{N} \sqrt{x^{2}_s+\varepsilon ^{2}}(\hat{y_s}-y_s) \nonumber \\&=\frac{1}{N} \sum _{s=1}^{N} \sqrt{x^{2}_s+\varepsilon ^{2}} (\hat{y_s}-(u x_s+r)) \nonumber \\&=\frac{1}{N} \sum _{s=1}^{N} \sqrt{x^{2}_s+\varepsilon ^{2}} ((\hat{y_s}-u x_s)-r) \end{aligned}$$

Where *s* represents the number of training samples. $\varepsilon$ is a very small constant. $\varepsilon$ is empirically set as $1e {-3}$. We utilized our model with the Charbonnier loss function instead of the $L_2$ loss to cope with outliers and improve MRI SR result accuracy, due to the loss is robust.

We also combined the GDL, which can directly penalize the differences of image gradient to sharpen the SR result. The GDL function is defined as follows:

4 $$\begin{aligned} L_{g d l}(y, \hat{y})&=\sum _{i, j}|| y_{i, j}-y_{i-1, j}|-| \hat{y}_{i, j}-\hat{y}_{i-1, j}||^{2}\nonumber \\&\quad +|| y_{i, j-1}-y_{i, j}| -| \hat{y}_{i, j-1} -\hat{y}_{i, j}||^{2} \end{aligned}$$

The overall GDL loss function is:

5 $$\begin{aligned} L_{g d l}(y, \hat{y})&=\frac{1}{N} \sum _{s=1}^{N} \sum _{i, j}|| y_{s_{i, j}}-y_{s_{i-1, j}}|-| \hat{y}_{s_{i, j}}-\hat{y}_{s_{i-1, j}}||^{2}\nonumber \\&\quad +|| y_{s_{i, j-1}-y_{i, j}}| -| \hat{y}_{s_{i, j-1}} -\hat{y}_{s_{i, j}}||^{2} \end{aligned}$$

Where |.| denotes the absolute value function.

Then the final combined loss is:

6 $$\begin{aligned} L_{combined} = L_{Charbonnier} + L_{g d l} \end{aligned}$$

## Dataset and training details

To verify the ability to reconstruct HR MRI slices of the brain, we applied our method on two adult-brain datasets (Kirby 21 and NAMIC) and eight clinical fetal MRIs.

### Dataset

#### Kirby 21 dataset

The Kirby 21 dataset^1^ contains the data of 21 volunteers who were all healthy, had no history of neurological conditions, and the dataset contained T1-weighted MRIs. The dataset was obtained using a 3-T MRI scanner (Achieva, Philips Healthcare, Best, Netherlands) with a sagittal view (FoV) of $240\times 204\times 256\ \hbox {mm}$ and a resolution of $1.0\times 1.0\times 1.2\ \hbox {mm}^3$.

#### NAMIC brain multimodality dataset

The NAMIC dataset (http://hdl.handle.net/1926/1687) was acquired using a 3-T General Electric (GE) device at Brigham and Women’s Hospital in Boston, MA. An eight-channel coil was employed to perform parallel imaging by using array spatial sensitivity encoding techniques^1^. The parameters of structural MRI were as follows: $\hbox {TR} = 7.4\ \hbox {ms}$, $\hbox {TE} = 3\ \hbox {ms}$, $25.6\ \hbox {cm}^2\ \hbox {FoV}$, and $\hbox {matrix} =256 \times 256$.

#### Clinical fetal MRI dataset

The eight clinical fetal MRI data was provided by the First Affiliated Hospital of Xi’an Jiaotong University. Images were continuously collected from September 2017 to October 2018 using GE 3.0-T MRI scanner (Discovery 750W; GE Medical system, Milwaukee, WI; $240\times 204\times 256\ \hbox {mm}$ FoV; 4-mm slice thickness; $\hbox {TE}=85$ ms) for fetal-head MRI. Eight pregnant volunteers used silent sequences, which contained silent T2 half-Fourier acquisition single-shot fast-spin-echo axial, sagittal, and coronal. These eight women underwent MRI scans because of health concerns. We performed the experiments by following the safety guidelines for MRI research. All patients signed informed consent forms, and the clinical protocol was approved by the Institutional Review Board of the First Affiliated Hospital of Xi’an Jiaotong University in Xi’an Shaanxi, China on February 25, 2019. The experimental data were completely de-identified, so that any related information of the subject cannot be retrieved.

### Training details

In order to validate our model, one tenth of the sections from each sequence of MRI were selected as validation data. We sliced Kirby21 and NAMIC datasets into 2D images. The total number of images is 1921. The whole images are split into 7:1:1:1 ratio as 1345 training, 192 for optimizing network weights, 192 for choosing hyper-parameters, and 192 for testing. We chose data from KKI2009-06 to KKI2009-42 in Kirby 21 to train the model. KKI2009-01, KKI2009-02, KKI2009-03, KKI2009-04, and KKI2009-05 were used for testing. We tested the model from case01011 to case01034 NAMIC. The remaining images were used for training. All eight fetal brain MRIs were used for testing. LR images were generated using a scale factor of two.

We initialized the network using the model of Lai^4^. The slope of leaky rectified linear units was $-0.2$. We padded zeros to make sure that the size of the feature map for each layer is the same as the input. And we trained the model by randomly sampling 64 patches whose sizes were all $128\times 128$. We set the momentum parameter to 0.9 and the weight decay to $1e{-4}$. The learning rate was initialized to $1e{-5}$ and decreased by a factor of two at every 50 epochs. We trained the original codes of the compared methods to calculate the runtime on the same computer with an Intel i7 processor (64-GB RAM) and Nvidia Tesla V100 graphics processor (16-GB Memory).
